# High TSH Level within Normal Range Is Associated with Obesity, Dyslipidemia, Hypertension, Inflammation, Hypercoagulability, and the Metabolic Syndrome: A Novel Cardiometabolic Marker

**DOI:** 10.3390/jcm8060817

**Published:** 2019-06-07

**Authors:** Yi-Cheng Chang, Shih-Che Hua, Chia-Hsuin Chang, Wei-Yi Kao, Hsiao-Lin Lee, Lee-Ming Chuang, Yen-Tsung Huang, Mei-Shu Lai

**Affiliations:** 1 Department of Internal Medicine, National Taiwan University Hospital, Taipei 100, Taiwan; b83401040@gmail.com (Y.-C.C.); chiahsuin123@yahoo.com.tw (C.-H.C.); leehsiaolin1103@gmail.com (H.-L.L.); leeming@ntu.edu.tw (L.-M.C.); 2 Department of Medicine, College of Medicine, National Taiwan University, Taipei 100, Taiwan; 3 Graduate Institute of Medical Genomics and Proteomics, National Taiwan University, Taipei 100, Taiwan; 4 Institute of Biomedical Science, Academia Sinica, Taipei 115, Taiwan; 5 Division of Endocrinology and Metabolism, Department of Internal Medicine, St. Martin De Porres Hospital, ChiaYi 600, Taiwan; tedshua@gmail.com; 6 Institute of Epidemiology and Preventive Medicine, College of Public Health, National Taiwan University, Taipei 100, Taiwan; 7 MJ Health Screening Center, Taipei 100, Taiwan; cutesttuna@gmail.com; 8 Graduate Institute of Molecular Medicine, National Taiwan University Hospital, Taipei 100, Taiwan; 9 Institute of Statistical Science, Academia Sinica, Taipei 115, Taiwan

**Keywords:** thyroid-stimulating hormone, cardiometabolic risks, metabolic syndrome, obesity, hypertension

## Abstract

(1) Background: Overt and subclinical hypothyroidism has been associated with increased cardiometabolic risks. Here we further explore whether thyroid function within normal range is associated with cardiometabolic risk factors in a large population-based study. (2) Methods: We screened 24,765 adults participating in health examinations in Taiwan. Participants were grouped according to high-sensitive thyroid-stimulating hormone (hsTSH) level as: <50th percentile (0.47–1.48 mIU/L, the reference group), 50–60th percentile (1.49–1.68 mIU/L), 60–70th percentile (1.69–1.94 mIU/L), 70–80th percentile (1.95–2.3 mIU/L), 80–90th percentile (2.31–2.93 mIU/L), and >90th percentile (>2.93 mIU/L). Cardiometabolic traits of each percentile were compared with the reference group. (3) Results: Elevated hsTSH levels within normal range were dose-dependently associated with increased body mass index, body fat percentage, waist circumferences, blood pressure, hemoglobin A1c (HbA1c), fasting insulin, homeostasis model assessment of insulin resistance (HOMA-IR), high homeostasis model of assessment of beta-cell (HOMA-β), triglycerides, total cholesterols, fibrinogen, and uric acids (*p*-for-trend <0.001), but not with fasting glucose levels. The association remained significant after adjustment of age, sex, and lifestyle. As compared to the reference group, subjects with the highest hsTSH percentile had significantly increased risk of being overweight (adjusted odds ratio (adjOR): 1.35), increased body fat (adjOR: 1.29), central obesity (adjOR: 1.36), elevated blood pressure (adjOR: 1.26), high HbA1c (adjOR: 1.20), hyperinsulinemia (adjOR: 1.75), increased HOMA-IR (adjOR: 1.45), increased HOMA-β (adjOR: 1.40), hypertriglyceridemia (adjOR: 1.60), hypercholesterolemia (adjOR: 1.25), elevated hsCRP (adjOR: 1.34), increased fibrinogen (adjOR: 1.45), hyperuricemia (adjOR: 1.47), and metabolic syndrome (adjOR: 1.42), but significant risk of low fasting glucose (adjOR: 0.89). Mediation analysis indicates that insulin resistance mediates the majority of the association between thyroid hormone status and the metabolic syndrome. (4) Conclusion: Elevated hsTSH within the normal range is a cardiometabolic risk marker associated with central obesity, insulin resistance, elevated blood pressure, dyslipidemia, hyperuricemia, inflammation, and hypercoagulability.

## 1. Introduction

Thyroid hormones exert profound effects on systemic metabolism, thermogenesis, and cardiovascular function [1]. Mice lacking thyroid hormone receptors develop hypotension, cold intolerance, and bradycardia [2]. Thyroid hormones enhance lipolysis in fat tissue and fatty acid oxidation in skeletal muscle, leading to improved insulin sensitivity in these tissues [3,4]. Thyroid hormones promote hepatic gluconeogenesis [3,5,6]. Therefore, clinically-overt hyperthyroid status is characterized by impaired glucose tolerance and elevated fasting glucose. Thyroid hormones exert complex effects on cholesterol metabolism in the liver [5,6,7]. Clinical hypothyroid status is associated with hypercholesterolemia due to impaired hepatic lipid clearance. Collectively, experimental and clinical studies show that thyroid hormones promote metabolic rate, thermogenesis, and weight loss, increase heart rate and blood pressure, reduce serum lipids levels, and improve insulin sensitivity in muscle and fat but elevate hepatic gluconeogenesis and fasting glucose.

Clinically-overt hypothyroidism is characterized by weight gain, cold intolerance, fluid retention, bradycardia, and hypercholesterolemia. Recent evidences demonstrate that subclinical hypothyroidism, defined as elevated thyroid-stimulating hormone (TSH) levels beyond normal range with normal thyroxine levels, is also associated with increased blood pressure, serum cholesterols [8,9,10,11], and cardiovascular disease risk [12,13,14]. In this study, we further explore whether TSH level within the normal limit is associated with cardiometabolic risk factors including central obesity, insulin resistance, high blood pressure, dyslipidemia, and inflammation in a large population-based study.

## 2. Materials and Methods

### 2.1. Study Population

Participants of this study were recruited from individuals who participated in a self-paying comprehensive health examination program offered by the MJ Health Management Institute in Taiwan between 2011 and 2016. The data used in this study were held and approved by MJ Health Management Institute, Taiwan. The authorization code is MJHRF2019007C. To comply with regulations related to the privacy of personal electronic data, the identity of every patient was delinked and all data was analyzed anonymously. The protocol was approved by the Research Ethics Committee in St. Martin De Porres Hospital in Taiwan. The ethics committee reference number is 18B-009. All methods were performed in accordance with the relevant guidelines and regulations of the Declaration of Helsinki.

### 2.2. Inclusion and Exclusion Criteria

Participants who had complete questionnaire information and complete metabolic, inflammation, and thyroid hormone assessment during 2011–2016 (*N* = 32,357) were included. We excluded (1) participants with age <20 years (*N* = 290); (2) those who reported to have received thyroid surgery or thyroid medications (*N* = 878); (3) those who already had hyperthyroidism (high-sensitive thyroid-stimulating hormone (hsTSH) level < 0.47 mIU/L) at the baseline (*N* = 1049); (4) those who already had overt hypothyroidism (*N* = 12); and (5) duplicated cases (*N* = 5363).

Participants were classified into those with high-sensitive thyroid-stimulating hormone (hsTSH) level <50th percentile (0.47–1.48 mIU/L), 50–60th percentile (1.49–1.68 mIU/L), 60–70th percentile (1.69–1.94 mIU/L), 70–80th percentile (1.95–2.3 mIU/L), 80–90th percentile (2.31–2.93 mIU/L), and >90th percentile (>2.93 mIU/L). Participants with TSH <50th percentile were used as the reference group. The normal upper limit of hsTSH is 5.0 mIU/L in the MJ Health Management Institute. The number of participantswith hsTSH higher than normal range (5.0 mIU/L) was only 325 (1.31%) in our study.

### 2.3. Data Collection

Self-reported questionnaire for lifestyle factors and past medical history were offered by each participant. Each participant undertook a standard panel of history taking, physical examinations, and laboratory tests. Details of the data collection were described elsewhere [15,16]. Overnight fasting blood were collected and analyzed. Alcohol consumption was defined by drinking more than 1 to 2 times a week. Physical inactivity was defined by exercising for less than 1 h a week. Cigarette smoking was defined by currently smoking more than 1 to 2 times a week.

The laboratory method for determination of TSH is a two-step immunoassay using chemiluminescent microparticle immunoassay (Abbott ARCHITECT I2000). In the first step, sample and anti-β TSH antibody-coated paramagnetic microparticles were combined. After washing, anti-α TSH acridinium labeled conjugate was added in the second step. The resulting chemiluminescent reaction was measured. The quality control requirement was a single sample of all control levels tested once every 24 h each day of use. The laboratory of MJ Health Management Institute has passed the ISO 9001:2000 requirements.

### 2.4. Definition of Cardiometabolic Risk Factors

Overweight was defined as body mass index (BMI) >24 kg/m^2^. Increased body fat (%) was defined as: body fat ≥20% in men and ≥25% in women with age ≤30 years and body fat ≥25% in men and ≥30% in women with age >30 years [17]. Increased waist circumference was defined as >90 cm in men and >80 cm in women. Elevated blood pressure was defined as systolic blood pressure >130 mmHg or diastolic blood pressure >85 mmHg. Elevated fasting glucose was defined as ≥100 mg/dL [18]. High HbA1c was defined as HbA1c >5.7%. Hyperinsulinemia was defined as fasting insulin ≥15 mIU/L. High homeostasis model of assessment of insulin resistance (HOMA-IR) was defined as HOMA-IR >3.0 mU/L·mM [19]. High homeostasis model of assessment of beta-cell (HOMA-β) was defined as HOMA-β >75th percentile of the study participants [20]. Hypertriglyceridemia was defined as fasting triglycerides ≥150 mg/dL [18]. Hypercholesterolemia was defined as total cholesterol >200 mg/dL. Low high-density lipoprotein cholesterol (HDL-C) was defined as ≤40 mg/dL in men and ≤50 mg/dL in women [18]. High low-density lipoprotein cholesterol (LDL-C) was defined as LDL-C ≥130 mg/dL. High triglyceride/HDL-C ratio was defined as TG/HDL-C >2.75 in men and >1.65 in women [21]. Elevated high sensitivity C-reactive protein (hsCRP) was defined as ≥3 mg/dL [21]. Elevated serum fibrinogen level was defined as >400 mg/dL [22]. Hyperuricemia was defined as >7.2 mg/dL in men and >6.0 mg/dL in women. Metabolic syndrome was defined according to the International Diabetes Federation (IDF) worldwide definition of the metabolic syndrome [18].Past history of diabetes mellitus was defined by self-reported medical history of diabetes mellitus or history of taking anti-diabetic drugs. Past history of hypertension was defined by self-reported medical history of hypertension or history of taking anti-hypertensive drugs. Past history of dyslipidemia was defined by self-reported medical history of dyslipidemia and history of taking lipid-lowering drugs. Past history of cardiovascular diseases was defined by self-reported medical history of cardiovascular diseases and history of taking cardiovascular drugs.

### 2.5. Statistical Analyses

Differences in the baseline characteristics of study participants across all hsTSH percentile groups were compared by using the trend test without and with adjustment for age, sex, smoking, alcohol drinking, and physical inactivity. The relation between dichotomous traits including increased adiposity, elevated blood pressure level, hyperglycemia, insulin resistance, dyslipidemia, inflammatory markers, and metabolic syndrome, and across all hsTSH categories, were further evaluated. Multinomial logistic regression was performed to calculate the adjusted odds ratio and 95% confidence intervals without and with controlling for age, sex, smoking, alcohol drinking, and physical inactivity. The optimal cut-off values of hsTSH for each cardiometabolic risk factor were calculated according to the Youden index (sensitivity + specificity − 1) [23]. Statistical analyses were performed using SAS version 9.4 (SAS Institute, Cary, NC, USA). A two-sided *p*-value of <0.05 was considered as statistically significant.

### 2.6. Mediation Analyses

We further investigated the mediation effects of insulin resistance (measured by HOMA-IR) linking low normal thyroid function (measured by hsTSH or free T4) to metabolic syndrome. More specifically, using mediation modeling, we evaluated the effect of low normal thyroid function on metabolic syndrome risk that is explained by insulin resistance. Mediation analyses were conducted using an existing method [24]. We briefly summarize the analyses in the following. First, we assume a joint model for the mediator and the outcome:

(1) $$\mathrm{logit}\;P(Y=1|A,M,X)=\beta_{X}^{T}X+\beta_{A}A+\beta_{M}M+\beta_{AM}A\times M$$

(2) $$\mathrm{logit}\;P(M=1|A,X)=\alpha_{X}^{T}X+\alpha_{A}A$$

where *Y*, *A*, *M*, and *X*, respectively, are the metabolic syndrome (i.e., the outcome), the hsTSH, HOMA-IR (i.e., the mediator), and the covariates, respectively. Direct and indirect effects of the thyroid function on the risk of metabolic syndrome in relation to insulin resistance can be calculated on the scale of risk difference:

(3) $$\mathrm{Direct}\;\mathrm{effect}=\Gamma \left(a_{1}=1,a_{2}=0,x\right)|\Gamma \left(a_{1}=0,a_{2}=0,x\right)$$

(4) $$\mathrm{Indirect}\;\mathrm{effect}=\Gamma \left(a_{1}=1,a_{2}=1,x\right)|\Gamma \left(a_{1}=1,a_{2}=0,x\right)$$

The direct and indirect effects have a causal interpretation provided that the adjustment of covariates satisfies the no-unmeasured confounding assumptions for identifiability: The indirect effect is the effect of the thyroid function on the risk of metabolic syndrome mediated by insulin resistance, and the direct effect is the effect not mediated through affecting insulin resistance. We also measured proportion of mediation as the logarithm of the indirect effect divided by the logarithm of the product of the direct and indirect effects. The proportion of mediation measured the percentage of the effect of the thyroid function on the risk of metabolic syndrome was mediated through insulin resistance, on the scale of log risk ratio. Confidence intervals of 95% of the measurement were calculated using bootstrapping.

## 3. Results

**Figure 1:**
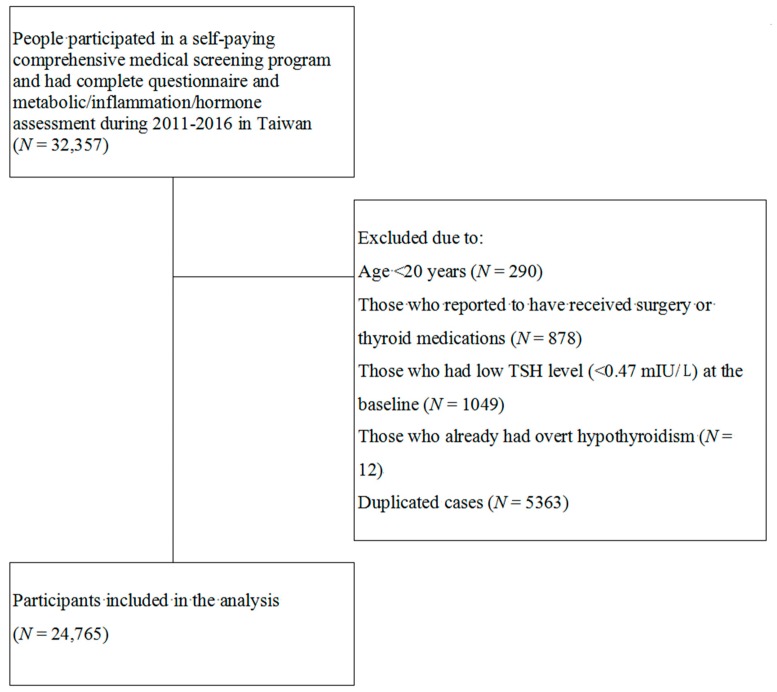
Study flow.

The study flow is depicted in Figure 1. After exclusion, a total of 24,765 participants were recruited. Their baseline characteristics are listed in Table 1.

We first examined continuous traits across different hsTSH percentile groups. The crude and adjusted *p*-for-trend is summarized in Table 2. As expected, free T4 levels decreased as hsTSH levels increased (*p*-for-trend < 0.0001). Increased hsTSH was associated with a higher proportion of females (*p*-for-trend < 0.0001) and older ages (*p*-for-trend < 0.0001). Increased hsTSH, even within normal range, was significantly associated with increased BMI, body fat percentage, waist circumference, systolic and diastolic blood pressure, HbA1c, fasting insulin, HOMA-IR, HOMA-β, triglycerides, total cholesterols, HDL-C, LDL-C, triglycerides/HDL-C ratio, fibrinogen, and uric acid, but was not associated with fasting glucose levels after adjustment for age, sex, smoking, alcohol drinking, and physical activity (Table 2). There was also a trend of positive association between hsTSH and hsCRP (*p*-for-trend = 0.083).

**Figure 2:**
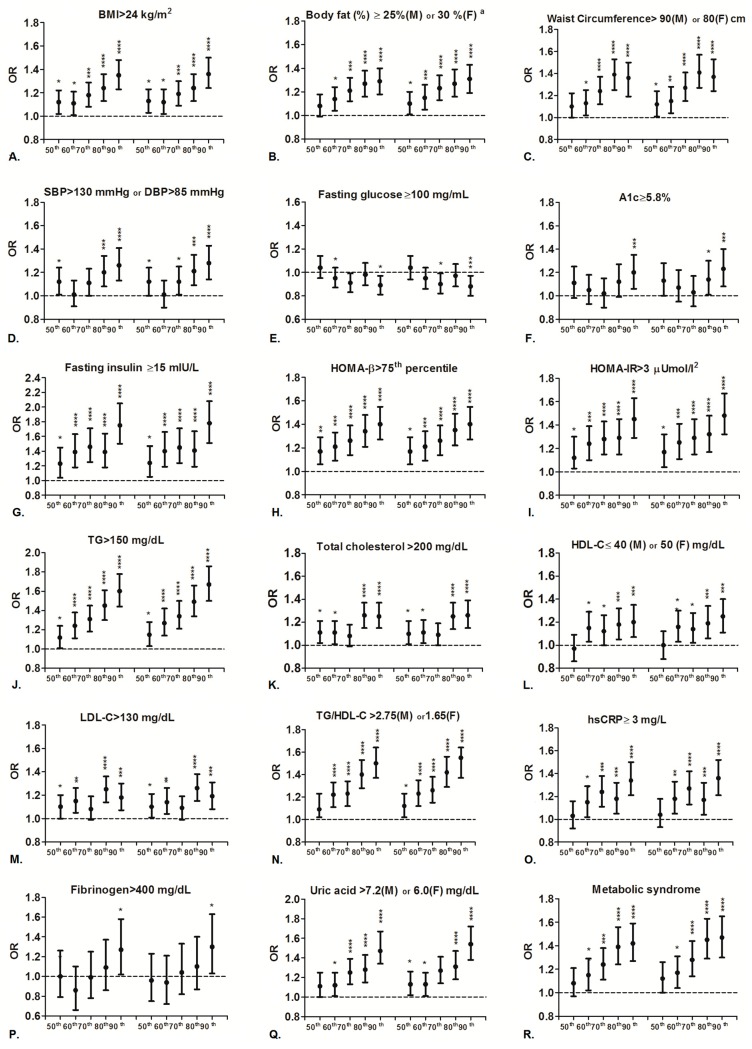
Crude (left panel) and adjusted (right panel) odds ratio for central obesity (**A**–**C**), elevated blood pressure (**D**), hyperglycemia (**E**,**F**), insulin resistance (**G**–**I**), dyslipidemia (**J**–**N**), inflammation (**O**), hypercoagulability (**P**), hyperuricemia (**Q**) and metabolic syndrome (**R**) among participants with different thyroid-stimulating hormone (TSH) levels (*N* = 24,765). * *p* < 0.05, ** *p* < 0.01, *** *p* < 0.001, **** *p* < 0.0001.

We examined the association between hsTSH levels and dichotomous cardiometabolic risk traits. The crude odds ratios (ORs) is listed in Supplementary Table S1 and Figure 2. Briefly, subjects with the highest hsTSH percentile had significantly increased ORs of overweight, high body fat percentage, central obesity, elevated blood pressure, high HbA1c, hyperinsulinemia, high HOMA-β, increased HOMA-IR, hypertriglyceridemia, hypercholesterolemia, high LDL-C, high triglycerides/HDL-C, elevated hsCRP, increased fibrinogen, hyperuricemia, and metabolic syndrome, but low fasting glucose and low HDL-C as compared with the reference group.There was no clear dose-responsive association of past history of hypertension, dyslipidemia, and cardiovascular disease with hsTSH levels. Only a trend of association of past history of diabetes mellitus with hsTSH was found.

We next calculated the optimal cut-off value of hsTSH for each metabolic phenotype according to the Youden index (Supplementary Table S1). The optimal cutoff of hsTSHranged from 1.147 mIU/L for overweight (BMI > 24 kg/m^2^) to 2.225 mIU/L for hypercoagulability (fibrinogen > 400 mg/dL). These resultssuggest the upper limit of “normal” hsTSHwith respect to cardiometabolic risk might need to be reset within this range.

The adjusted ORs are summarized in Table 3 and Figure 2. Similarly, subjects with the highest hsTSH percentile had significantly increased risk of overweight (adjusted OR (adjOR): 1.36, 95% confidence interval (CI) = (1.24, 1.50); *p* < 0.0001), high body fat percentage (adjOR: 1.31, 95% CI = (1.19–1.43), *p* < 0.0001), central obesity (adjOR: 1.37, 95% CI = (1.24–1.53), *p* < 0.0001), elevated blood pressure (adjOR:1.28, 95% CI = (1.14–1.43), *p* < 0.0001), high HbA1c (adjOR: 1.23, 95% CI = (1.08–1.40), *p* = 0.0015), hyperinsulinemia (adjOR: 1.78, 95% CI = (1.51–2.08), *p* < 0.0001), high HOMA-β (adjOR:1.40, 95% CI = (1.27–1.55), *p* < 0.0001), increased HOMA-IR (adjOR: 1.48, 95% CI = (1.32–1.67), *p* < 0.0001), hypertriglyceridemia (adjOR: 1.67, 95% CI = (1.50–1.86), *p* < 0.0001), hypercholesterolemia (adjOR: 1.26, 95% CI = (1.15–1.39), *p* < 0.0001), high LDL-C (adjOR:1.19, 95% CI = (1.08–1.31), *p* = 0.0005), high triglycerides/HDL-C ratio (adjOR: 1.55, 95% CI = (1.41–1.70) *p* < 0.0001), elevated hsCRP (adjOR: 1.36, 95% CI = (1.21–1.52), *p* < 0.0001), increased fibrinogen (adjOR: 1.30, 95% CI = (1.03–1.63), *p* = 0.02), hyperuricemia (adjOR: 1.54, 95% CI = (1.38–1.72), *p* < 0.0001), and metabolic syndrome (adjOR: 1.47, 95% CI = (1.30–1.65), *p* < 0.0001), but had significant risk of low fasting glucose (adjOR: 0.88, 95% CI = (0.80–0.97), *p* = 0.0091) and low HDL-C (adjOR: 1.25, 95% CI = (1.11–1.40), *p* = 0.0002) as compared with the reference group.

Since insulin resistance had been proposed as the unifying underlying cause of metabolic syndrome [18], we next conducted mediation analyses to model the relationship between thyroid hormone status, insulin resistance, and metabolic syndrome. Specifically, we studied the effect of hsTSH or free T4 on the risk of metabolic syndrome mediated through insulin resistance as measured by HOMA-IR (Supplementary Table S2). In comparison to patients with lower hsTSH levels than the median, those with higher hsTSH levels than the median had a higher risk of metabolic syndrome mediated by HOMA-IR, with a significant effect (indirect effect) measured on the scale of risk difference being 0.63% (95% confidence interval = (0.45%, 0.83%); *p*-value < 0.0001). The direct effect of high hsTSH (versus low hsTSH) on the risk of metabolic syndrome not mediated through HOMA-IR also revealed a positive effect, with a risk difference of 0.74% (95% CI = (0.42%, 1.05%), *p* < 0.0001) (Supplementary Table S2). Of the overall hsTSH effect on metabolic syndrome, 46.0% were mediated through HOMA-IR, suggesting that insulin resistance is an important mediator for the effect of thyroid function on metabolic syndrome (Supplementary Table S2).

In addition to hsTSH, we also investigated the effect of lower free T4 levels than the median level, which showed a similar pattern (Supplementary Table S2). In comparison to those with high free T4 (greater than the median level), patients with low free T4 were more likely to develop metabolic syndrome, either mediated by HOMA-IR or not, with indirect and direct effects on the scale of risk difference being 0.54% (95% CI = (0.36%, 0.74%), *p* < 0.0001) and 0.26% (95% CI = (−0.08%, 0.60%), *p* = 0.066), respectively. The proportion of the free T4 effect on metabolic syndrome mediated by HOMA-IR was 67.2%, even higher than that of hsTSH (Supplementary Table S2). Taken together, mediation analyses suggest that the effect of thyroid hormone status on metabolic syndrome is significantly mediated by insulin resistance and such a mediation explained the majority of the effect (46.0% for hsTSH; 67.2% for free T4).

## 4. Discussion

In this large population-based study involving 24,765 subjects, we found that increased hsTSH levels, even within the normal range, is associated with increased risk of central obesity, insulin resistance, elevated blood pressure, hyperglycemia, hyperlipidemia, hyperuricemia, inflammation, hypercoagulability, and metabolic syndrome, suggesting hsTSH as a novel cardiometabolic risk marker. The association was probably mediated through insulin resistance. To our knowledge, this study is the largest study to investigate the association of hsTSH within normal levels in a range of comprehensive cardiometabolic traits using mediation analysis.

Consistently, in a cross-sectional study involving 2703 euthyroid participants from Netherlands, TSH levels were positively associated with insulin resistance and triglycerides levels [25]. In another study involving 1283 euthyroid Chinese, those with TSH levels between 1.91 to 4.80 mIU/L had significantly larger waist circumferences, higher BMI, and a trend of higher serum triglycerides, but a trend of lower prevalence of hyperglycemia than those with TSH between 0.30 to 0.99 mIU/L [26]. In a study involving 2760 Korean euthyroid women, TSH levels were positively associated with waist circumference, blood pressure, and triglycerides, but no fasting glucose or HDL-C [27]. Another study involving 2153 euthyroid Bulgarians showed that the highest TSH quartiles were associated with higher triglycerides, lower HDL-C, and metabolic syndrome, but not with abdominal obesity, hypertension, or diabetes/prediabetes in comparison to the lowest quartiles [28]. In a study involving 201 Italian women, TSH was positively associated with waist circumference [29]. In a study involving 1333 euthyroid Germans, TSH in the upper normal range was associated with higher BMI, higher triglycerides, and metabolic syndrome, but not fasting glucose in comparison to those with TSH in the lower normal range [30]. Another study involving 2771 euthyroid Mexicans showed that TSH was positively associated with waist circumference, systolic blood pressure, total cholesterols, LDL-C, triglycerides, fasting insulin, and HOMA-IR, but not with HDL-C, diastolic pressure, and fasting glucose [31]. In another study involving 3755 euthyroid Iranians, TSH was positively associated with waist circumference, triglycerides, but not with total cholesterol, LDL-C, HDL-C, systolic and diastolic blood pressure, and fasting blood glucose [32]. Collectively, in most studies, higher TSH level is associated with central obesity, elevated blood pressure, increased triglycerides, and insulin resistance, but not with fasting glucose. The mechanism by which elevated TSH is associated with reduced fasting glucose is probably related to the thyroid hormones’ action to promote hepatic gluconeogenesis [3,5,6]. It is well-established that the thyroid hormone stimulates gluconeogenesis through up-regulating phosphoenolpyruvate carboxykinase (PEPCK) [33] and increasing sympathetic input to the liver [6]. Rodents with the mutant thyroid receptor display reduced hepatic glucose output [34]. This is consistent with our findings, and other clinical findings, that elevated TSH is associated with lower fasting glucose. Whether this finding will affect the overall cardiovascular risk is currently unknown.

In an analysis targeted to analyze the association of TSH with serum lipids in 30,656 euthyroid Norwegians, there was a linear and significant increase in total cholesterols, LDL-C, and triglycerides with increasing TSH [35]. In another study targeted to analyze the association of TSH with serum lipids in 3664 euthyroid Chinese, TSH was linearly positivity associated with total cholesterols and triglycerides [36]. In another study analyzing the association of TSH with serum lipids, TSH was significantly associated with total cholesterols, triglyceride, and LDL-C, but not with HDL-C, in 7270 euthyroid Koreans [37]. In another analysis targeted to investigate the association between TSH and blood pressure in 30,728 euthyroid Norwegians, increased TSH was significantly associated with increased blood pressure. Higher TSH (3.0–3.5 mIU/L) was associated with increased risk of hypertension in men and women [38].

We observed increased TSH levels within the normal range were significantly associated with increased hsCRP. There is little information mentioning the association between TSH and hsCRP. In a Brazilian study of 12,284 subjects with THS within euthyroid and subclinical hypothyroidism, TSH levels were not associated with CRP [39]. In a Turkey study involving 77 subclinical hypothyroid cases and 50 euthyroid controls, subclinical hypothyroidism was associated with elevated hsCRP [40].

We also observed increased TSH levels were significantly associated with hypercoagulability. Consistently, in a study of 959 French subjects, free T4 levels were inversely correlated with serum fibrinogen level [41]. Conversely, another population-based study in 3804 Germans found that low serum TSH was associated with high fibrinogen levels [42]. Our results strongly support that elevated TSH within the euthyroid range is associated with low-grade inflammation and hypercoagulability, probably related to increased adiposity.

In contrast to our findings, two large cross-sectional studies found a negative association between TSH with measures of obesity [43,44]. A study involving 5998 participants from general Korean population showed that TSH levels were negatively associated with waist circumference and HDL-C, and positively with triglycerides [42]. In another study involving 13,496 euthyroid Koreans, TSH as negatively related to BMI, HDL-C, fasting glucose, and HbA1c [44]. The reason for the discrepancy of TSH association with measures of obesity is not currently known.

Instead of TSH, several studies investigated the association of thyroxine levels with cardiometabolic risk factors. A study involving 303 euthyroid Greeks showed that free T4 levels were negatively associated with subcutaneous fat mass [45]. Another study involving 44,196 euthyroid Koreans showed that normal high free T4 was associated with lower waist circumference [46]. Intriguingly another study involving 941 euthyroid Belgian men showed free T4 levels were positively associated with whole body fat mass and trunk fat mass, but negatively associated with whole body lean mass and radius muscle mass [47]. In conclusion, low normal thyroxine may be associated with increased fat mass.

Using mediation analysis, we found that insulin resistance mediates the association between the metabolic syndrome and free T4 (67.2%) or hsTSH (46.0%). This is consistent with the established action of thyroxine to promote insulin sensitivity of fat and skeletal muscle in experimental models. Because of the difference in measurement techniques, serum hsTSH is a much more sensitive marker than free T4 for assessing thyroid status. Our study showed that hsTSH is a highly sensitive marker of thyroid status associated with metabolic syndrome through the thyroxine’s action on insulin resistance.

Our study has some unique strengths. First, this is the largest study investigating the association of high normal TSH with the most comprehensive coverage of cardiometabolic risk factors and the mechanism using mediation analyses. In addition, the lab tests are performed as screening tests rather than for a clinical indication, which prevents the confounding by indication. Our detailed questionnaires coverage of lifestyle enables adjustments for multiple confounding factors and mediation analysis.

Our study has several limitations. First, a longitudinal follow-up study is required in the future since this is a cross-sectional study. Second, although we adjusted various lifestyle factors, residual confounding factors may still exist. Third, the participants are recruited from voluntary health examinations but not from random sampling. Therefore, the risk estimation may not be applicable for the general population.

## 5. Conclusions

A linear dose-dependent association was found between hsTSH within the normal range with central obesity, dyslipidemia, elevated blood pressure, inflammation, hypercoagulability, and metabolic syndrome, but not with fasting glucose, in a large population. The association is probably mediated through the thyroid hormone’s action on insulin sensitivity. Our results suggest high hsTSH within the euthyroid range is a novel cardiometabolic risk marker.

## Figures and Tables

**Table 1:** Baseline characteristics of participants (*N* = 24,765).

Characteristics	Mean (SD)
Male (number, %)	11,811 (47.69)
Age (years)	44.43 (12.41)
High-sensitive thyroid stimulating hormone (hsTSH) (mIU/L)	1.74 (1.08)
Free T4 (µU/mL)	1.06 (0.13)
Body mass index (kg/m^2^)	23.77 (3.97)
Body fat (%)	27.55 (7.31)
Waist circumference (cm)	78.57 (10.95)
Systolic blood pressure (mmHg)	115.64 (17.80)
Diastolic blood pressure (mmHg)	73.86 (11.21)
Fasting glucose (mg/dL)	104.69 (23.30)
Hemoglobin A1c (%)	5.34 (0.78)
Fasting Insulin (mIU/L)	7.94 (6.61)
Homeostasis model assessment of insulin resistance (HOMA-IR) (mU/L·mM)	2.13 (2.29)
Homeostasis model assessment of beta-cell (HOMA-β) (mU/L/mM)	72.44 (51.91)
Triglycerides (mg/dL)	118.23 (105.00)
Total cholesterol (mg/dL)	199.06 (36.46)
High-density lipoprotein cholesterol (mg/dL)	58.57 (15.11)
Low-density lipoprotein cholesterol (mg/dL)	117.95 (33.55)
Triglycerides/High-density lipoprotein cholesterol ratio	2.33 (3.27)
High-sensitive C-reactive protein (mg/L)	1.97 (4.38)
Fibrinogen (mg/dL)	289.64 (56.42)
Uric acid (mg/dL)	5.68 (1.54)
Alcohol consumption (number, %)	4151 (17.39)
Cigarette smoking (number, %)	4004 (16.75)
Physical inactivity (number, %)	15,538 (65.77)
History of diabetes mellitus (number, %)	1017 (4.11)
History of hypertension (number, %)	2736 (11.07)
History of dyslipidemia (number, %)	769 (3.11)
History of cardiovascular diseases (number, %)	1211 (4.9)

SD: Standard deviation; alcohol consumption was defined by drinking more than 1 to 2 times a week; physical inactivity was defined by exercising for less than 1 h a week. Cigarette smoking was defined by currently smoking more than 1 to 2 times a week.

**Table 2:** Characteristics of study participants with different hsTSH levels (*N* = 24,765).

Characteristic	hsTSH Level (mIU/L)						Crude *p* Value for Trend	Adjusted *p* Value for Trend *
	<50th Percentile 0.47–1.48	50–60th Percentile 1.49–1.68	60–70 thPercentile 1.69–1.94	70–80th Percentile 1.95–2.3	80–90th Percentile 2.31–2.93	>90th Percentile >2.93		
Number	12,004	2676	2510	2646	2506	2423	-	-
Male (number, %)	6161 (51.32)	1320 (49.33)	1203 (47.93)	1216 (45.96)	1025 (40.90)	886 (36.57)	<0.0001	-
Age (year)	44.05 (12.07)	44.34 (12.42)	44.96 (12.3)	44.61 (12.68)	44.97 (12.72)	45.09 (13.43)	<0.0001	-
Free T4 (ng/dL)	1.068 (0.134)	1.063 (0.118)	1.058 (0.114)	1.056 (0.1165)	1.0522 (0.119)	1.0365 (0.121)	<0.0001	<0.0001
BMI (kg/m2)	23.60 (3.78)	23.88 (4.08)	23.88 (3.96)	24 (4.16)	23.89 (4.18)	24.02 (4.24)	<0.0001	<0.0001
Body fat (%)							<0.0001	<0.0001
Man	24.38 (5.82)	24.74 (6.19)	25.02 (5.98)	25.06 (6.37)	25.13 (6.19)	25.01 (6.47)		
Women	29.59 (7.07)	30.30 (7.63)	30.08 (7.11)	30.56 (7.53)	30.96 (7.76)	31.33 (7.85)		
Waist circumference (cm)							0.46	<0.0001
Men	84.40 (9.07)	85.02 (9.58)	85.54 (9.50)	85.59 (10.03)	86.01 (9.80)	85.95 (10.33)		
Woman	72.17 (8.43)	72.63 (8.68)	72.60 (8.52)	73.15 (8.73)	73.32 (8.86)	73.97 (9.74)		
Systolic blood pressure (mmHg)	115.1 (17.34)	116.4 (18.04)	115.7 (17.57)	116.14 (18.27)	115.95 (18.34)	116.35 (18.77)	0.0383	<0.0001
Diastolic blood pressure (mmHg)	73.76 (11.08)	74.10 (11.35)	73.89 (11.14)	74.03 (11.44)	73.7 (11.19)	74.02 (11.5)	0.88	<0.0001
Fasting glucose (mg/dL)	104.78 (23.19)	104.69 (22.24)	104.6 (21.77)	104.48 (23.78)	104.51 (24)	104.74 (25.19)	0.78	0.24
HbA1c (%)	5.33 (0.77)	5.33 (0.75)	5.33 (0.75)	5.35 (0.77)	5.36 (0.81)	5.36 (0.81)	0.034	0.0002
Fasting Insulin (mIU/L)	7.78 (7.02)	7.98 (5.84)	8.01 (5.43)	7.99 (5.26)	8.14 (5.86)	8.39 (8.29)	0.0002	<0.0001
HOMA-IR (mU/L·mM )	2.09 (2.50)	2.13 (1.78)	2.15 (1.82)	2.14 (1.89)	2.18 (2.02)	2.27 (2.73)	0.0015	<0.0001
HOMA-β(mU/L/mM )	70.58 (54.02)	72.99 (54.39)	72.74 (41.95)	73.42 (42.39)	74.93 (47.05)	77.12 (60.83)	<0.0001	<0.0001
TG (mg/dL)	113.67 (95.68)	117.25 (93.75)	122.97 (111.16)	121.91 (147.42)	124.42 (103.92)	126.59 (98.4)	<0.0001	<0.0001
Total cholesterol (mg/dL)	197.44 (35.66)	199.81 (37.65)	199.6 (35.04)	199.32 (37.71)	202.27 (37.84)	202.1 (37.19)	<0.0001	<0.0001
HDL-C (mg/dL)	58.54 (15.24)	58.67 (14.81)	58.09 (15)	57.92 (14.48)	59.16 (15.42)	59.2 (15.23)	0.031	<0.0001
LDL-C (mg/dL)	117.03 (33.25)	118.72 (33.92)	118.33 (32.96)	118.49 (34.51)	119.6 (33.96)	118.96 (33.68)	0.0088	<0.0001
TG/HDL-C	2.24 (3.06)	2.28 (2.34)	2.45 (3.01)	2.45 (5.53)	2.42 (2.66)	2.47 (2.57)	0.0008	<0.0001
hs-CRP (mg/L)	1.94 (4.95)	1.93 (3.82)	2.01 (4.06)	2.05 (3.95)	1.95 (3.14)	2.08 (3.7)	0.1850	0.0883
Fibrinogen (mg/dL)	287.67 (56.23)	289.8 (56.54)	289.84 (56.89)	291.94 (55.99)	292.8 (56.71)	293.24 (56.57)	<0.0001	0.0188
Uric acid (mg/dL)	5.67 (1.52)	5.71 (1.57)	5.71 (1.56)	5.73 (1.58)	5.65 (1.53)	5.68 (1.57)	0.6316	<0.0001

* Adjusted for age, sex, smoking, alcohol drinking, and physical inactivity. hsTSH: High-sensitive thyroid-stimulating hormone. BMI: body mass index; HbA1c: hemoglobin A1c; HOMA-IR; Homeostasis Model Assessment of Insulin Resistance; HOMA-β: Homeostasis Model Assessment of Beta-Cell; TG: triglycerides; HDL-C: high-density lipoprotein cholesterol; LDL-C: low-density lipoprotein cholesterol; hs-CRP: high-sensitive C-reactive protein.

**Table 3:** The adjusted odds ratio for increased adiposity, elevated blood pressure, dyslipidemia, insulin resistance, hyperglycemia, inflammatory markers, and metabolic syndrome among participants with different high-sensitive thyroid-stimulating hormone (hsTSH)levels (*N* = 24,765).

Multinomial Logistic Regression										
Variable	hsTSH Level (mIU/L)									
	1.49–1.68 vs. 0.47–1.48		1.69–1.94 vs. 0.47–1.48		1.95–2.3 vs. 0.47–1.48		2.31–2.93 vs. 0.47–1.48		>2.93 vs. 0.47–1.48	
	adjOR (95% CI)	*p* Value	adjOR (95% CI)	*p* Value	adjOR (95% CI)	*p* Value	adjOR (95% CI)	*p* Value	adjOR (95% CI)	*p* Value
BMI > 24 kg/m^2^	1.13 (1.03, 1.23)	0.01	1.12 (1.02, 1.23)	0.02	1.19 (1.09, 1.30)	0.0002	1.24 (1.13, 1.36)	<0.0001	1.36 (1.24, 1.50)	<0.0001
Body fat (%): ≤30 years: male ≥20%; female ≥25% >30 years: male ≥25%; female ≥30%	1.10 (1.01, 1.20)	0.03	1.15 (1.05, 1.26)	0.0022	1.23 (1.13, 1.34)	<0.0001	1.27 (1.16, 1.39)	<0.0001	1.31 (1.19, 1.43)	<0.0001
Waist circumference: Men > 90 cm; women > 80 cm	1.12 (1.01, 1.24)	0.03	1.15 (1.04, 1.28)	0.0082	1.27 (1.15, 1.41)	<0.0001	1.41 (1.27, 1.57)	<0.0001	1.37 (1.24, 1.53)	<0.0001
Systolic blood pressure > 130 mmHg or Diastolic blood pressure > 85 mmHg	1.12 (1.00, 1.24)	0.04	1.01 (0.90, 1.13)	0.86	1.12 (1.01, 1.25)	0.03	1.21 (1.09, 1.35)	0.0006	1.28 (1.14, 1.43)	<0.0001
Fasting glucose ≥ 100 mg/dL	1.04 (0.94, 1.14)	0.45	0.95 (0.86, 1.04)	0.26	0.90 (0.82, 0.99)	0.03	0.97 (0.88, 1.07)	0.54	0.88 (0.80, 0.97)	0.0091
HbA1c ≥ 5.8 %	1.13 (1.00, 1.28)	0.05	1.07 (0.95, 1.22)	0.28	1.03 (0.91, 1.17)	0.68	1.14 (1.01, 1.30)	0.04	1.23 (1.08, 1.40)	0.0015
Fasting insulin ≥ 15 mIU/L	1.24 (1.05, 1.47)	0.01	1.40 (1.19, 1.66)	<0.0001	1.45 (1.24, 1.71)	<0.0001	1.41 (1.19, 1.67)	<0.0001	1.78 (1.51, 2.08)	<0.0001
HOMA-β > 75th percentile	1.17 (1.06, 1.29)	0.0022	1.21 (1.09, 1.34)	0.0003	1.26 (1.14, 1.39)	<0.0001	1.35 (1.22, 1.49)	<0.0001	1.40 (1.27, 1.55)	<0.0001
HOMA-IR > 3.0 (mU/L·mM)	1.17 (1.04, 1.32)	0.0082	1.25 (1.11, 1.41)	0.0002	1.29 (1.15, 1.45)	<0.0001	1.32 (1.17, 1.48)	<0.0001	1.48 (1.32, 1.67)	<0.0001
TG ≥ 150 mg/dL	1.15 (1.03, 1.28)	0.01	1.27 (1.14, 1.42)	<0.0001	1.34 (1.21, 1.50)	<0.0001	1.49 (1.34, 1.66)	<0.0001	1.67 (1.50, 1.86)	<0.0001
Total Cholesterol > 200 mg/dL	1.10 (1.01, 1.21)	0.03	1.11 (1.02, 1.22)	0.02	1.09 (1.00, 1.19)	0.06	1.25 (1.14, 1.37)	<0.0001	1.26 (1.15, 1.39)	<0.0001
HDL-C: men ≤ 40 or women ≤ 50 mg/dL	1.00 (0.88, 1.12)	0.96	1.16 (1.03, 1.30)	0.01	1.14 (1.02, 1.28)	0.02	1.19 (1.06, 1.34)	0.0037	1.25 (1.11, 1.40)	0.0002
LDL-C ≥ 130 mg/dL	1.10 (1.01, 1.21)	0.04	1.14 (1.04, 1.26)	0.0051	1.09 (0.99, 1.19)	0.08	1.26 (1.15, 1.38)	<0.0001	1.19 (1.08, 1.31)	0.0005
TG/HDL-C: men > 2.75 or women > 1.65	1.12 (1.02, 1.23)	0.01	1.23 (1.12, 1.35)	<0.0001	1.26 (1.15, 1.38)	<0.0001	1.42 (1.29, 1.56)	<0.0001	1.55 (1.41, 1.70)	<0.0001
hs-CRP ≥ 3 mg/dL	1.04 (0.93, 1.18)	0.49	1.18 (1.05, 1.33)	0.0057	1.27 (1.13, 1.42)	<0.0001	1.17 (1.04, 1.32)	0.0087	1.36 (1.21, 1.52)	<0.0001
Fibrinogen > 400 mg/dL	0.96 (0.75, 1.23)	0.75	0.94 (0.72, 1.21)	0.61	1.04 (0.82, 1.33)	0.73	1.10 (0.87, 1.40)	0.43	1.30 (1.03, 1.63)	0.02
Uric acid: men > 7.2 or women > 6.0 mg/dL	1.13 (1.02, 1.26)	0.03	1.13 (1.01, 1.27)	0.03	1.27 (1.14, 1.41)	<0.0001	1.31 (1.18, 1.47)	<0.0001	1.54 (1.38, 1.72)	<0.0001
Metabolic syndrome	1.12 (1.00, 1.26)	0.05	1.17 (1.04, 1.31)	0.01	1.28 (1.14, 1.44)	<0.0001	1.45 (1.29, 1.63)	<0.0001	1.47 (1.30, 1.65)	<0.0001
History of diabetes mellitus	1.07 (0.85, 1.34)	0.54	1.01 (0.80, 1.27)	0.93	1.05 (0.83,1.31)	0.68	1.16 (0.93, 1.45)	0.18	1.32 (1.05, 1.63)	0.013
History of hypertension	1.15 (0.99, 1.32)	0.056	1.11 (0.95, 1.28)	0.16	1.03 (0.89, 1.20)	0.68	1.19 (1.03, 1.38)	0.018	1.08 (0.92, 256)	0.35
History of dyslipidemia	1.14 (0.88, 1.45)	0.32	1.21 (0.94, 1.55)	0.12	1.30 (1.03, 1.65)	0.03	1.03 (0.79, 1.35)	0.81	1.17 (0.91, 1.52)	0.23
History of cardiovascular diseases	1.21 (0.99, 1.47)	0.05	1.02 (0.82, 1.25)	0.88	0.96 (0.78, 1.19)	0.72	1.28 (1.06, 1.56)	0.012	1.18 (0.96, 1.45)	0.11

* Adjusted for age, sex, smoking, alcohol drinking, and physical inactivity. Past history of diabetes mellitus was defined by self-reported medical history of diabetes mellitus or history of taking anti-diabetic drugs. Past history of hypertension was defined by self-reported medical history of hypertension or history of taking anti-hypertensive drugs.Past history of dyslipidemia was defined by self-reported medical history of dyslipidemia and history of taking lipid-lowering drugs. Past history of cardiovascular diseases was defined by self-reported medical history of cardiovascular diseases and history of taking cardiovascular drugs. BMI: body mass index; HbA1c: hemoglobin A1c; HOMA-IR; Homeostasis Model Assessment of Insulin Resistance; HOMA-β: Homeostasis Model Assessment of Beta-Cell; TG: triglycerides; HDL-C: high-density lipoprotein cholesterol; LDL-C: low-density lipoprotein cholesterol; hs-CRP: high-sensitive C-reactive protein; adjOR: Adjusted odds ratio; CI: Confidence interval.

## Supplementary Materials

The following are available online at https://www.mdpi.com/2077-0383/8/6/817/s1, Table S1: The crude odds ratio for increased adiposity, elevated blood pressure, dyslipidemia, insulin resistance, hyperglycemia, inflammatory markers, and metabolic syndrome among participants with different TSH levels (*N* = 24,765); Table S2: Mediation analysis modeling the relationship between thyroid function, insulin resistance, and metabolic syndrome.
