# Supplementary material for: High TSH Level within Normal Range Is Associated with Obesity, Dyslipidemia, Hypertension, Inflammation, Hypercoagulability, and the Metabolic Syndrome: A Novel Cardiometabolic Marker
Source: J Clin Med. 2019 Jun 7;8(6):817. doi: 10.3390/jcm8060817 (PMC6616443; doi:10.3390/jcm8060817)
Supplement: Supplementary file 1 [file jcm-08-00817-s001.pdf]

Supplementary Table 1 The crude odds ratio for increased adiposity, elevated blood pressure, dyslipidemia, insulin resistance, hyperglycemia, inflammatory markers, and metabolic syndrome among participants with different TSH levels (N = 24,765)

	Multinominal logistic regression											
	hsTSH 1.49-1.68 vs 0.47-1.48 (mIU/L)		hsTSH 1.69-1.94vs 0.47-1.48 (mIU/L)		hsTSH 1.95-2.3vs 0.47-1.48 (mIU/L)		hsTSH 2.31-2.93vs 0.47-1.48 (mIU/L)		hsTSH >2.93vs 0.47-1.48 (mIU/L)		Optimal hsTSH cut-off	Youden Index
**Variable**	**Crude OR (95%CI)**	**P value**	**CrudeOR (95%C)**	**P value**	**Crude OR (95%CI)**	**P value**	**Crude OR (95%CI)**	**P value**	**Crude OR (95%CI)**	**P value**		
**BMI>24 kg/m2**	1.12 (1.02, 1.22)	0.01	1.11 (1.01, 1.21)	0.03	1.18 (1.08, 1.29)	0.0003	1.24 (1.13, 1.36)	<0.0001	1.35 (1.23, 1.48)	<0.0001	1.147	0.0294
**Body fat (%) ≤30yrs: male≥20%; female ≥25% >30yrs: male≥25%; female ≥30%)**	1.08 (0.99, 1.18)	0.07	1.14 (1.04, 1.24)	0.0039	1.21 (1.12, 1.32)	<0.0001	1.27 (1.16, 1.38)	<0.0001	1.29 (1.18, 1.40)	<0.0001	1.643	0.0469
**Waist circumference Men>90 cm Women>80 cm**	1.10 (1.00, 1.22)	0.06	1.13 (1.02, 1.25)	0.02	1.24 (1.12, 1.37)	<0.0001	1.39 (1.25, 1.53)	<0.0001	1.36 (1.22, 1.50)	<0.0001	1.199	0.0525
**Systolic blood pressure> 130 mmHg or Diastolic blood pressure > 85 mmHg**	1.12 (1.01, 1.24)	0.04	1.01 (0.91, 1.13)	0.81	1.11 (1.00, 1.23)	0.06	1.20 (1.08, 1.34)	0.0008	1.26 (1.13, 1.41)	<0.0001	1.379	0.0255
**Fasting glucose≥100 mg/dL**	1.04 (0.95, 1.14)	0.38	0.95 (0.87, 1.04)	0.29	0.91 (0.83, 0.99)	0.04	0.98 (0.89, 1.08)	0.69	0.89 (0.81, 0.97)	0.01	1.943	0.0255
**HbA1c≥5.8**	1.11 (0.98, 1.25)	0.10	1.05 (0.93, 1.18)	0.46	1.02 (0.90, 1.15)	0.75	1.12 (0.99, 1.27)	0.06	1.20 (1.06, 1.35)	0.0041	1.531	0.0276
**Fasting insulin≥15 mIU/L**	1.23 (1.04, 1.45)	0.02	1.39 (1.18, 1.63)	<0.0001	1.46 (1.25, 1.71)	<0.0001	1.39 (1.18, 1.64)	<0.0001	1.75 (1.50, 2.05)	<0.0001	1.259	0.0879
**HOMA-β>75th percentile**	1.17 (1.06, 1.29)	0.0017	1.21 (1.09, 1.33)	0.0002	1.26 (1.14, 1.39)	<0.0001	1.34 (1.21, 1.48)	<0.0001	1.40 (1.27, 1.55)	<0.0001	1.359	0.0497
**HOMA-IR >3.0**	1.16 (1.03, 1.30)	0.01	1.24 (1.10, 1.39)	0.0003	1.28 (1.15, 1.43)	<0.0001	1.29 (1.15, 1.45)	<0.0001	1.45 (1.29, 1.63)	<0.0001	1.538	0.0528
**TG≥150 mg/dL**	1.12 (1.01, 1.24)	0.04	1.24 (1.11, 1.38)	<0.0001	1.31 (1.18, 1.45)	<0.0001	1.45 (1.30, 1.61)	<0.0001	1.60 (1.44, 1.78)	<0.0001	1.230	0.0614
**Total Cholesterol >200 mg/dL**	1.11 (1.02, 1.21)	0.02	1.11 (1.01, 1.21)	0.02	1.08 (0.99, 1.18)	0.07	1.26 (1.15, 1.37)	<0.0001	1.25 (1.15, 1.37)	<0.0001	1.286	0.0413
**HDL-C: men≤40;women≤50 mg/dL**	0.97 (0.86, 1.09)	0.58	1.15 (1.03, 1.29)	0.02	1.12 (1.00, 1.26)	0.04	1.18 (1.05, 1.32)	0.005	1.20 (1.07, 1.35)	0.002	2.159	0.0193
**LDL-C≥130 mg/dL**	1.10 (1.00, 1.20)	0.04	1.15 (1.05, 1.26)	0.002	1.08 (0.99, 1.19)	0.08	1.25 (1.14, 1.36)	<0.0001	1.18 (1.07, 1.30)	0.0006	1.271	0.0298
**TG/HDL-C: men>2.75 women>1.65**	1.09 (1.00, 1.12)	0.05	1.22 (1.11, 1.33)	<0.0001	1.23 (1.12, 1.34)	<0.0001	1.40 (1.28, 1.53)	<0.0001	1.50 (1.37, 1.64)	<0.0001	1.723	0.0649
**hs-CRP≥3 mg/dL**	1.03 (0.92, 1.16)	0.57	1.15 (1.02, 1.29)	0.02	1.24 (1.11, 1.38)	0.0002	1.18 (1.05, 1.32)	0.005	1.34 (1.20, 1.50)	<0.0001	1.777	0.0499

**Fibrinogen>400 mg/dL**	1.00 (0.79, 1.26)	0.99	0.86 (0.66, 1.10)	0.23	0.99 (0.78, 1.25)	0.93	1.09 (0.86,1.37)	0.48	1.27 (1.02, 1.58)	0.04	2.225	0.0473
**Uric acid: men>7.2 women>6.0 mg/dL**	1.11 (1.00, 1.23)	0.05	1.12 (1.01, 1.25)	0.04	1.25 (1.13, 1.39)	<0.0001	1.28 (1.15, 1.43)	<0.0001	1.47 (1.34, 1.67)	<0.0001	1.620	0.0382
**Metabolic syndrome**	1.08 (0.97, 1.21)	0.18	1.15 (1.02, 1.29)	0.02	1.24 (1.11, 1.38)	0.0002	1.39 (1.24, 1.56)	<0.0001	1.42 (1.27, 1.59)	<0.0001	1.784	0.0522

Supplementary Table 2 Mediation analysis modeling the relationship between thyroid function, insulin resistance, and metabolic syndrome

(A) Modeling hypothesis and regression parameters

Model: hsTSH/FT4→Insulin Resistance(HOMA-IR)→ Metabolic Syndrome

**Model**	**Y**	**X**		**Z**		**M**		**Interaction**
**1**	Metabolic Syndrome [0/1]	Age [continuous] Gender [0/1]	Smoking [0/1=current]	High hsTSH	[0/1 cutoff=50th %]	HOMA-IR	[0/1 cutoff=3]	Z*M
**2**	Metabolic Syndrome [0/1]	Age [continuous] Gender [0/1]	Smoking [0/1=current]	LowFT4	[0/1 cutoff=50th %]	HOMA-IR	[0/1 cutoff=3]	Z*M

(B) Parameter estimates of models

**Model**	**Δ Direct effect**		**Δ Indirect effect**		**ρ Direct effect**		**ρ Indirect effect**		**Proportion of mediation**
	**Estimate (95% CI)**	**p-value**	**Estimate (95% CI)**	**p-value**	**Estimate (95% CI)**	**p-value**	**Estimate (95% CI)**	**p-value**	**Estimate (95% CI)**
**1**	0.0074 (0.0042, 0.0105)	<0.0001	0.0063 (0.0045, 0.0083)	<0.0001	1.0079 (1.0045, 1.011)	<0.0001	1.0067 (1.0047,1.0088)	<0.0001	0.46 (0.34, 0.62)
**2**	0.0026 (-0.0008,0.0060)	0.066	0.0054 (0.0036, 0.0074)	<0.0001	1.0028 (0.99, 1.0064)	0.065	1.0058 (1.0038,1.0079)	<0.0001	0.67 (0.43, 1.17)

Two-sided p-value and 95% CI were derived from bootstrap for 10,000 times

Y, A, M, and X represent the metabolic syndrome (i.e. outcome), the high-sensitive thyroid-stimulating hormone (hsTSH),Homeostasis Assessment of Insulin Resistance (HOMA-IR) (i.e. the mediator) and the covariates. Direct and indirect effects of the thyroid function on the risk of metabolic syndrome in relation to insulin resistance are calculated.
